# Incidence and predictors of loss to follow-up among Ethiopian children on antiretroviral therapy: a systematic review and meta-analysis

**DOI:** 10.1186/s12889-023-17333-9

**Published:** 2024-01-13

**Authors:** Molla Yigzaw Birhanu, Getamesay Molla Bekele, Getasew Yirdaw, Bekele Simegn Demissie, Genanew Kassie Getahun, Selamawit Shita Jemberie

**Affiliations:** 1 Department of Public Health, College of Health Sciences, Debre Markos University, P.O. Box 269, Debre Markos, Ethiopia; 2 Department of Gynecology and Obstetric, School of Medicine, Debre Markos University, Debre Markos, Ethiopia; 3 Department of Environmental Health, College of Health Sciences, Debre Markos University, Debre Markos, Ethiopia; 4 Department of Public Health, St.Lideta College of Health Science and Business, Addis Ababa, Ethiopia; 5 Department of Public Health, Menelik II Medical and Health Science College, Addis Ababa, Ethiopia; 6 Department of Midwifery, College of Health Sciences, Debre Markos University, Debre Markos, Ethiopia

**Keywords:** LTFU, Incidence, Predictors, Children, ART, Ethiopia

## Abstract

**Introduction:**

Loss of follow-up (LTFU) from ART regular follow-up is one of the key acknowledged causes for the development of ART-resistant virus strains currently. It becomes a major weakness for the successful implementation of HIV care and treatment programs mainly in Sub-Saharan Africa but also globally. About 20—40% of children on ART loss their regular ART follow-up annually. Because of the inconsistency of the prior publications' findings, policymakers, programmers, and healthcare providers find it difficult to intervene. Hence, this study was conducted to provide a pooled incidence and identify the predictors of LTFU among children on ART in Ethiopia.

**Methods:**

Articles were searched from PubMed/ MEDLINE, CINAHL, EMBASE, Google Scholar, and Science Direct, as well as organizational records and websites. This review included both retrospective and prospective follow-up studies published in English. The data were extracted using Microsoft Excel and exported into Stata™ Version 17.0 for further processing and analysis. The presence of heterogeneity was assessed using forest plots with the I^2^ test. To identify the source of heterogeneity subgroup analysis, meta-regression, publication bias, and sensitivity analysis were computed. The pooled incidence of LTFU was estimated using a random effects meta-analysis model with the DerSimonian-laired method. To identify the predictors, a 95% confidence interval with relative risk was used to declare the presence or absence of an association.

**Results:**

In this systematic review and Meta-analysis, nine studies with a total of 3336 children were included. The pooled incidence of LTFU from ART was 5.83 (95% CI: 3.94, 7.72) per 100 children-years of observation with I^2^: 83% & *p*-value < 0.001. Those children who were from rural were had a 1.65 (95% CI: 1.06, 2.52) times higher chance of getting LTFU when compared with their counterparts. Children who had poor ART adherence had a 2.03 (95% CI: 1.23, 3.34) times higher chance of experiencing LTFU of ART than children having good ART adherence.

**Conclusions:**

Among Ethiopian children on ART, one out of 167 had the risk of experiencing LTFU. Being rural dwellers and having poor ART adherence were the identified predictors of LTFU. Close follow-up and phone message text should be used to have good ART adherence among rural dwellers to meet the predetermined goal of ART.

**Supplementary Information:**

The online version contains supplementary material available at 10.1186/s12889-023-17333-9.

## Introduction

Human Immunodeficiency Virus (HIV) deteriorates the human immune system and makes the body vulnerable to secondary and opportunistic infections [1]. The progression of HIV infection in children is especially rapid in the absence of HIV care antiretroviral therapy [2]. Antiretroviral therapy (ART) determined impact and outcome could be achieved when the children on ART had good adherence to regular follow-ups [3].

Globally, it has been estimated that out of 1.7 million children living with HIV, 65% of them received Anti-retroviral Therapy (ART), and 57% of them had viral suppression at the end of 2021 [4]. As ART coverage grows, a rise in LTFU has been observed in many African ART programs, with children faring the worst. Loss to follow-up (LTFU) of ART is defined as failing to engage in the continuum of care for 90 days (3 months) after the last scheduled appointment due to their wishes or beliefs or barriers to continued access to care [5]. Here we can understand that, LTFU is a major obstacle to the success of HIV treatment in meeting the UNAIDS 95–95-95 goals in 2025 & and ending the HIV epidemic by 2030 [6]with an estimated 20–40% of patients experiencing loss to follow-up in Sub-Saharan Africa countries [7] and 14 -28% globally [8]. Children who have failed antiretroviral medication have an increased number of side effects like expansion of drug-resistant viral strains and mortality [9]. The emergence of drug-resistant strains, which results in the transmission of drug-resistant strains to the population, has devastating consequences, rendering future therapeutic interventions ineffective [10] and narrowing the subsequent possible alternatives, as well as affecting the success of HIV treatment in meeting the Joint United Nations Program on HIV/AIDS (UNAIDS) 95–95-95 goals in 2025 ending HIV epidemic by 2030 [11].

ART failure may be secondary to LTFU, and it is a growing issue (such as increasing ART accessibility) that threatens to undermine much of the work that has been prioritized for patients, the community, and the country at large.

Despite the fact that several primary studies on the incidence and predictors of LTFU among Ethiopian children on ART have been conducted, this study was conducted to estimate the pooled incidence and predictors of LTFU among Ethiopian children on ART due to the presence of conflicting (inconsistent) findings, which makes programmers, policymakers, and healthcare professionals difficult.

The findings of this study will be important for designing mechanisms for interventions like prevention to improve the quality of life of children.

### Research question

What are the incidence and predictors of loss to follow-up among Ethiopian children on ART?

### Condition

Loss to follow-up of ART.

### Context

Ethiopia.

### Population

Ethiopian children on ART.

## Methods

### Study design

Systematic review and Meta-analysis.

### Study setting

Ethiopia is a Federal Democratic Republic with nine regional states (Afar, Amhara, Benishangul-Gumuz, Gambella, Harari, Oromia, Somali, Southern Nations Nationalities and People's Region, and Tigray) and two city administrations (Addis Ababa and Dire Dawa). It has a total area of 1,100,000 km2 and is divided into zones, which are further subdivided into districts, which are further subdivided into kebeles, the lowest administrative divisions [12]. Ethiopia, with a population of approximately 112 million people, is Africa's second most populous country (56,010, 000 females and 56, 069, 000 males in 2019) [13].

### Participants

All cohort research articles conducted on incidence and predictors of loss to follow-up among Ethiopian children and published in English.

### Data source and searching strategy

This review was reported using the Strengthening the Reporting of Observational Studies in Epidemiology (STROBE) guideline [14], (Additional file 1). We conducted a systematic search of electronic databases (PubMed/MEDLINE, CINAHL, EMBASE, Google Scholar, and Science Direct) for previous research related with our topic of interest. In addition to the databases, articles were also found by searching the reference lists of eligible studies. Two authors worked independently on the search (MYB and SSJ). Endnote X9 was used to retrieve and manage studies found after conducting a systematic search. Of the search engine, the following is the example used in PubMed database searches: (((((((HIV infected children[Text Word]) OR (Children living with HIV[Text Word])) OR (children on ART[Text Word])) OR (HIV infected Adolesecent[Text Word])) OR (Adolescents living with HIV[Text Word])) OR (HIV infected children[MeSH Terms])) AND (((Ethiopia[Text Word]) OR (Federal Democratic Republic of Ethiopia[Text Word])) OR (Ethiopia[MeSH Terms]))) AND (((((((Loss to follow up[Text Word]) OR (withdraw[Text Word]))) OR (New occurrence[Text Word])) OR (incidence[Text Word])) OR (predictor*s[Text Word])) OR (Loss to follow up[MeSH Terms]))." The search strategy began on December 25, 2022, and ended on January 12, 2023.

### Eligibility criteria

#### Included criteria

All cohort studies conducted on incidence and predictors of loss to follow-up among Ethiopian children on ART and Published in English were eligible.

### Excluded

Articles were excluded when they did not have the outcome variable and those articles having the same title but different study designs.

### Screening procedure/ study size

Two authors (MYB and SA) independently screened all titles/abstracts found in electronic databases. Two authors (MYB and SSJ) independently screened the full text of screened and included articles for title and abstract. Disagreements during the screening of the title and full length were resolved through discussion in the presence of the third author (GMB).

### Quality assessment (risk of bias)

The Newcastle–Ottawa Quality Assessment Scale for cohort study (NOQAS) [15] was used to evaluate the quality of the included primary studies based on selection (Representativeness of the exposed cohort, Selection of the non-exposed cohort, Ascertainment of exposure, and Demonstration that outcome of interest was not present at start of study), Comparability(based on the design or analysis controlled for confounders), and outcome (Assessment of outcome, Was follow-up long enough for outcomes to occur, Adequacy of follow-up of cohorts). During the quality assessment of articles, all included articles were declared as having good quality because the articles scored 3 stars in the selection domain AND 2 stars in the comparability domain AND 2 stars in the outcome/exposure domain.

### Data extraction

The data were extracted using the data extraction Checklist prepared from a Microsoft Excel spreadsheet. To ensure consistency, three authors (MYB, SSJ, and BE) extracted data independently using a predefined extraction checklist. After the source of the disagreements was identified, disagreements between or among authors were resolved through discussion. The incidence of LTFU of ART among children, the study region, year of publication, sample size, follow-up period, and the first author's name were all the extracted data from the primary article during extraction.

### Outcome variable and measures

The primary outcome of interest was the pooled incidence of LTFU from ART. It was calculated by considering the incidence of primary studies and its standard error using the random effects model through the DerSmonian-laired method and I^2^ tests. The second outcome of interest was the pooled predictors of LTFU which was identified using a binary meta-regression model with a 95% confidence level and the strength of association has been presented using relative risk. It was again calculated after the log transformation of the primary studies' effect size had been further computed. First, the RR of the primary studies was transformed into logRR to get the actual effect size and its standard error was computed using lnlogRR. Hereafter, the binary meta-regression model was fitted considering the logRR and lnlogRR to identify the predictors of first-line ART failure among Ethiopian school children. Finally, the association between variables was presented using RR with a 95% CI.

### Data management and analysis

For further analysis, the extracted data were exported to Stata™ Version 17.0 software. The pooled incidence of loss to follow-up from ART was estimated using the random effects model with the DerSimonian laired method. The standard errors were calculated from the reported estimates and population denominators using a binomial distribution assumption. The presence of heterogeneity between studies was determined using the Cochran-Q test and quantified using I-square statistics. The level of heterogeneity was classified as low (I^2^:0—25), moderate(I^2^: 25—50), or high (I^2^: ≥ 50) [19. For the first time, the presence of heterogeneity was checked using a forest plot test through a fixed effects model with inverse variance method and revealed the presence of heterogeneity. Thereafter, a forest plot test through the random-effect model with the DerSimonian and Laird method was computed [16]. After getting heterogeneity, subgroup analysis, publication bias, sensitivity analysis, and meta-regression were performed to identify the source of heterogeneity but not explained. The study regions, publication year (before 2021 vs. after 2021), sample size (above mean vs. below mean), and follow-up period (< 8 vs. > 8 years) were used to conduct a subgroup analysis. To determine the presence of publication bias after the funnel plot, Egger's linear regression test and trim and fill analysis were used to declare the effect of small studies [17]. Finally, the findings depending on the objectives of the study were presented in the form of tables and figures.

## Results

### Search results

**Fig. 1:**
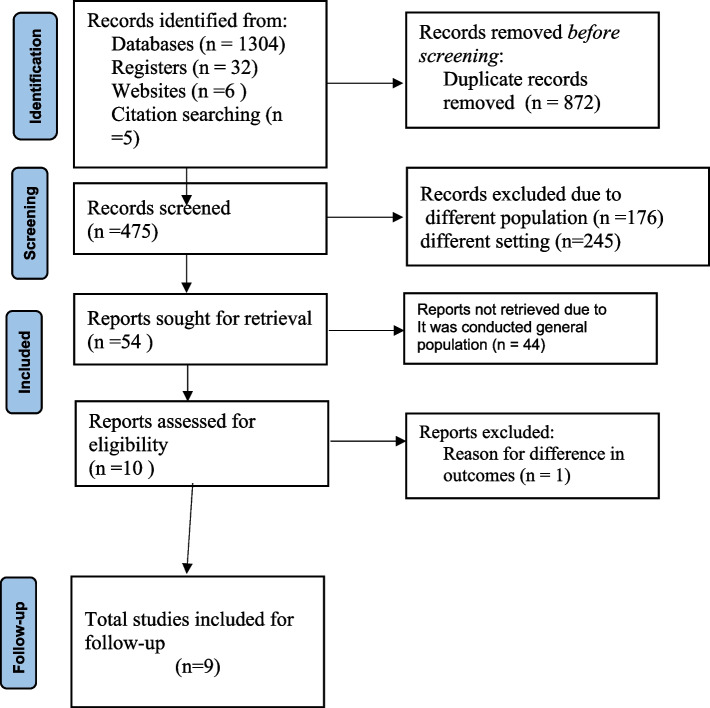
STROBE flow diagram of the included studies for LTFU from ART among Ethiopian children

A total of 1304 studies were discovered through electronic database searches on PubMed/ MEDLINE, CINAHL, EMBASE, Google Scholar, and Science Direct, as well as organizational records and websites. Approximately 872 articles were excluded due to duplication, 245 articles were excluded due to differences in study setting/context [17–22], 133 articles were excluded due to differences in study population [23–29], 45 articles were excluded due to the study conducted on the general population [18, 30–32]. Finally, 9 cohort studies were identified for inclusion and followed for the current Systematic Review and Meta-analysis (Fig. 1).

### Characteristics of the included articles

In Ethiopia, about nine studies qualified for inclusion and analysis, involving a total of 3336 children on ART which were published up to 2022. These studies were conducted in three regions and one city administration. Among the included studies, about 5 [33–37] of them were in the Amhara region, 1 [38] in the Oromia region, 1 [39] in SNNPR, and 2 [40] in Addis Ababa. The studies with the smallest and largest sample sizes, 254 and 533, were conducted in SNNPR and Addis Abeba respectively, followed by a study conducted at Amhara region with a sample size of 448. The included studies' follow-up periods ranged from 2 to 14 years, with 287.7 to 1555.56 child-years of ART failure-free observation (Table 1).

**Table 1:** The characteristics of the included studies among Ethiopian children on ART

Sn	Authors	Publication year	Region	cases	Sample size	PYO	Incidence	Follow-up period	Quality assessment
1	Tiruye Menshw et al. [33]	2021	Amhara	101	448	1603.17	6.3	10	Good
2	TAMENE FETENE etal [34]	2018	Addis Ababa	46	533	317.6	14.5	5	Good
3	Chalachew Adugna Wubneh et al. [35]	2020	Amhara	24	402	800	6	5	Good
4	Yitbarek Tenaw Hibstie et al. [37]	2019	Amhara	70	408	1555.56	4.5	14	Good
5	Kirubel Biweta Bimer et al. [39]	2020	SNNPR	70	254	1357.6	5.2	6	Good
6	Mulatu Biru et al. [40]	2018	Addis Ababa	8	304	287.7	2.8	2	Good
7	Ermias Sisay Chanie et al. [41]	2022	Amhara	76	357	1590.1	4.8	13	Good
8	Assefa Washo Bankere et al. [38]	2022	Oromia	43	269	1,299	3.3	5	Good
9	Selam Fisiha Kassa et al. [36]	2020	Amhara	79	361	1280.8	6.2	11	Good

### The pooled incidence of loss to follow-up

**Fig. 2:**
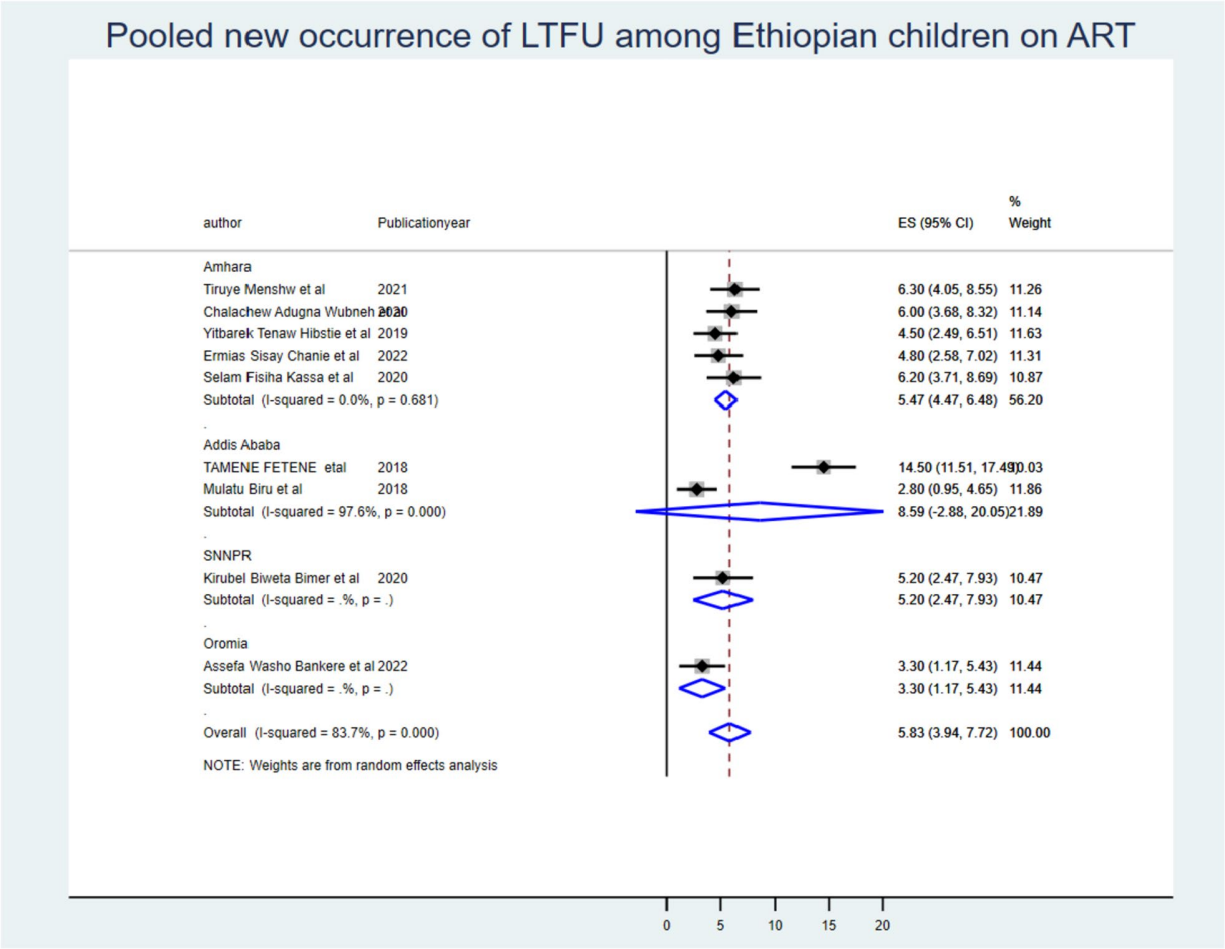
The pooled new occurrences of loss to follow-up among Ethiopian children on ART

The incidence of LTFU from ART among Ethiopian children was 5.83 (95% CI: 3.94, 7.72) per 100 children-years of observation with I^2^: 83.7% & *P*-value < 0.001. When we looked at it by region, Addis Ababa city administration had 8.59 (95% CI: -2.88, 20.05) incidence of LTFU among children, SNNPR had 5.20 (95% CI: 2.47, 7.93), and Oromia region had 3.30 (95% CI: 1.17, 5.43) per 100 children-years of ART failure free observation (Fig. 2).

### Subgroup meta-analysis

**Fig. 3:**
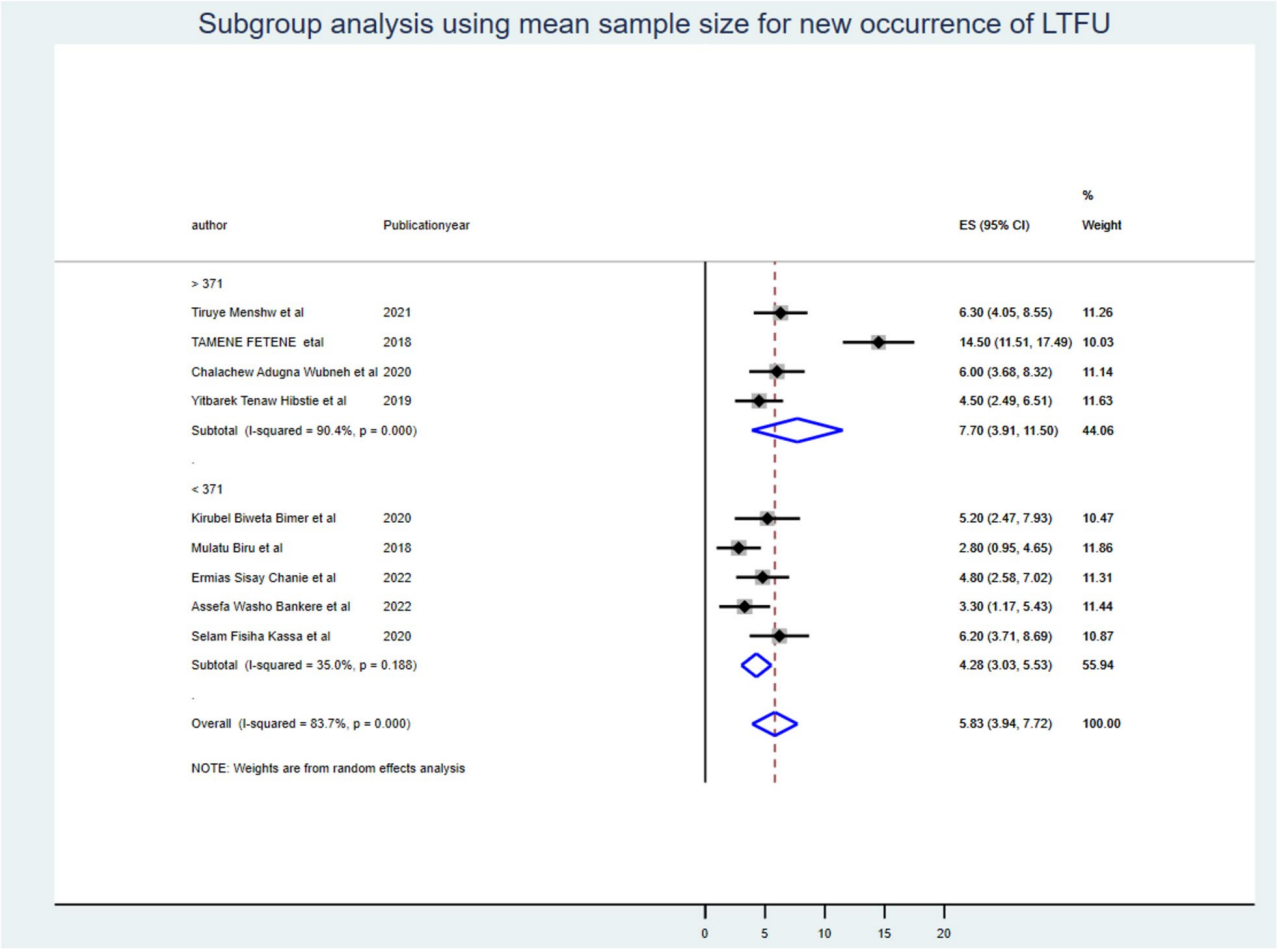
Subgroup analysis using mean sample size for LTF

**Fig. 4:**
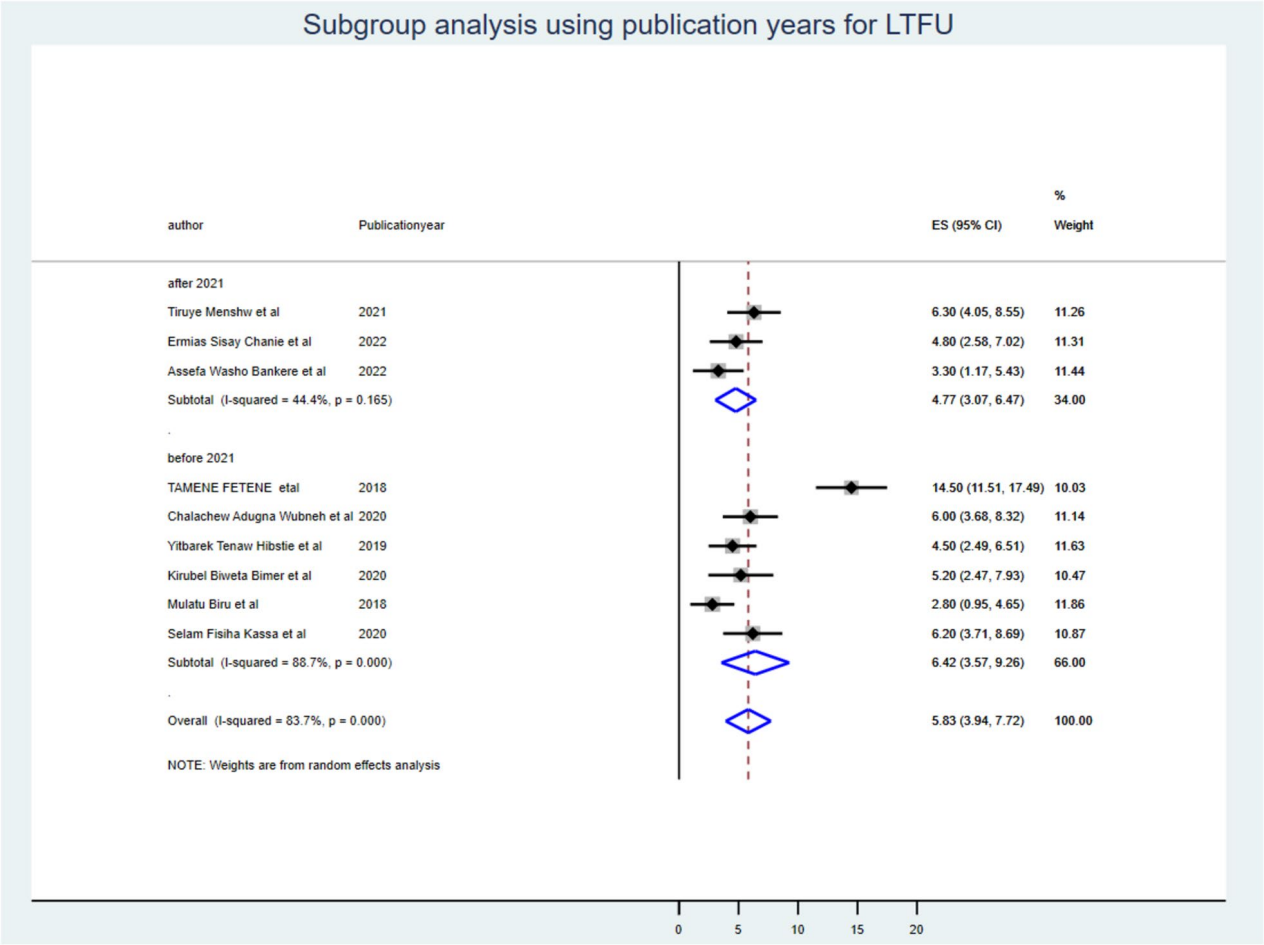
Subgroup analysis using publication years for LTFU

**Fig. 5:**
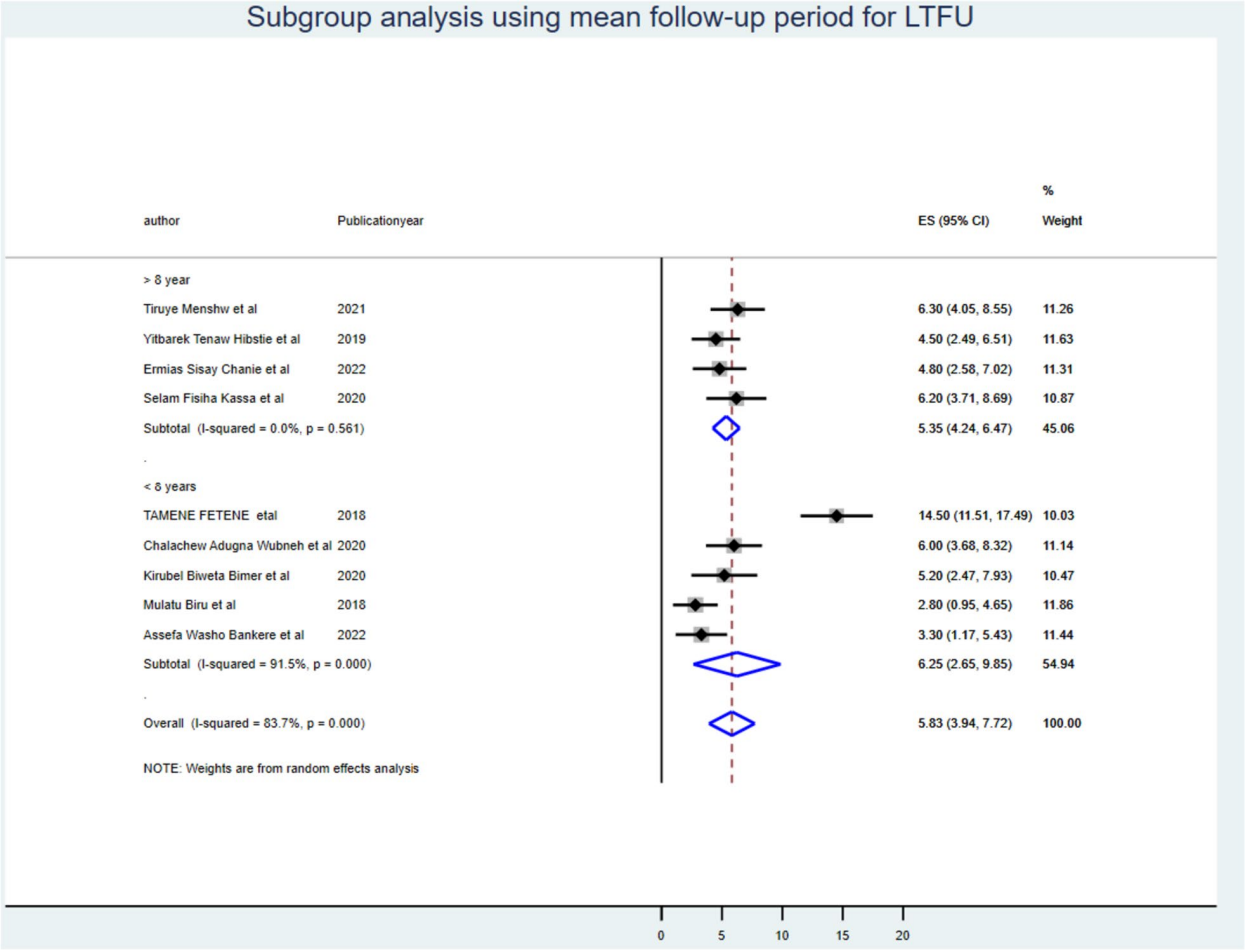
Subgroup analysis using mean follow-up period for LTFU

Despite the presence of strong evidence supporting the existence of heterogeneity (using sample size for those whose sample size was less than the mean (< 371) I^2^ = 35.19 with *P*-value < 0.19 & > 371 I^2^ = 90.40 with *P*-value < 0.001 (Fig. 3), publication year (those published before 2021 I^2^ = 88.00 with *P*-value < 0.00 & after 2021 I^2^ = 44.17 with *P*-value < 0.001) (Fig. 4), and length of follow-up period ( those conducted less than the mean follow-up years (< 8 years) I^2^ = 91.40 with *P*-value < 0.001, & those conducted greater than the mean follow-up years (> 8 years) I^2^ = 0.00 with *P*-value < 0.56) (Fig. 5), no sources of heterogeneity were identified using subgroup Meta-analysis.

### Meta-regression

The meta-regression is the extension of subgroup analysis conducted to identify the source of heterogeneity using continuous variables. Thus, the publication year and sample size were used as covariates in random-effects meta-regression. The analysis revealed that sample size (*P*-value = 0.43) and publication year (*P*-value = 0.88) did not affect heterogeneity (Table 2).

**Table 2:** Meta-regression using publication year and sample size for LTFU in Ethiopian children

Logrr	Coefficient	Std. err	t	P >|t|	[95% conf. interval]
Sample size	0.01	0.01	0.84	0.43	-0.01 0.02
Publication year	0.04	0.27	0.15	0.88	-0.62 0.70
Constant	-83.52	545.71	-0.15	0.88	-1418.83 1251.78

### Publication bias (Bias detection)

**Fig. 6:**
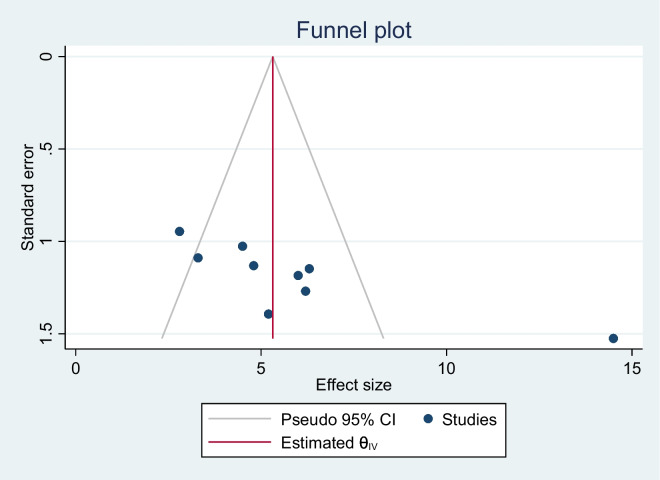
Funnel plot to check publication bias of LTFU among Ethiopian children on ART

The presence or absence of publication bias was initially determined using a funnel plot, and the distribution of the studies in the funnel plot was asymmetrical, indicating that the small studies did not affect the heterogeneity (Fig. 6). As well known, the funnel plot is a subjective measure for assessing publication bias; so, the Egger linear regression test was used to confirm the presence of publication bias objectively, and it demonstrated that small studies had an effect on the existence of heterogeneity. Hence, Trim and fill analysis was performed as a tiebreaker to obtain a definitive conclusion on the presence of publication bias, and it yielded 5.85, which is consistent with the funnel plots estimate. Finally, we declared that small studies did not affect the existence of heterogeneity among studies (Table 3).

**Table 3:**
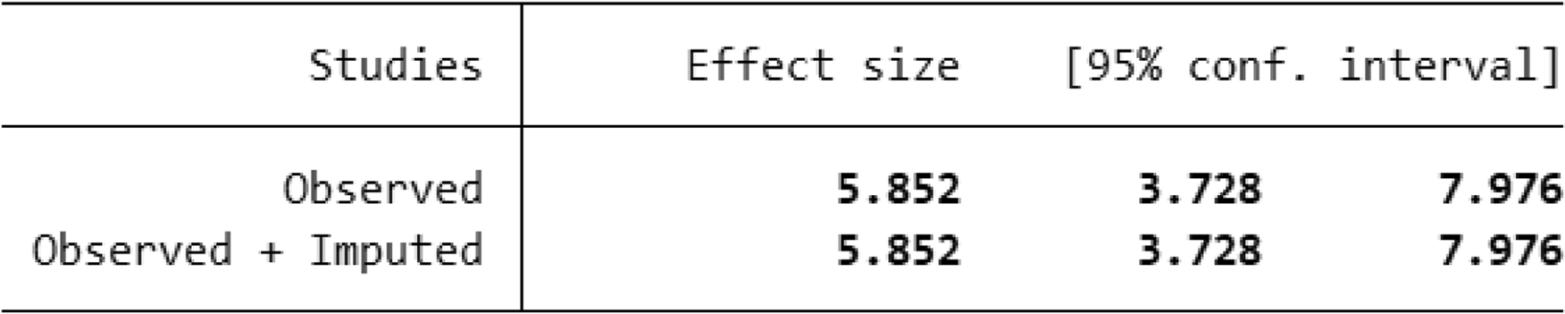
Heterogeneity check through conducting publication bias check

### Predictors of LTFU among Ethiopian children

Baseline WHO stage (including six studies), adherence (including three studies), disclosure status (including two studies), parents' educational status, baseline CD4 count, and parental status were used to predict the new occurrence of LTFU of ART in Ethiopian children. As a result, being a rural resident and having poor ART adherence were identified as significant predictors for the incidence of LTFU from ART among Ethiopian Children.

**Fig. 7:**
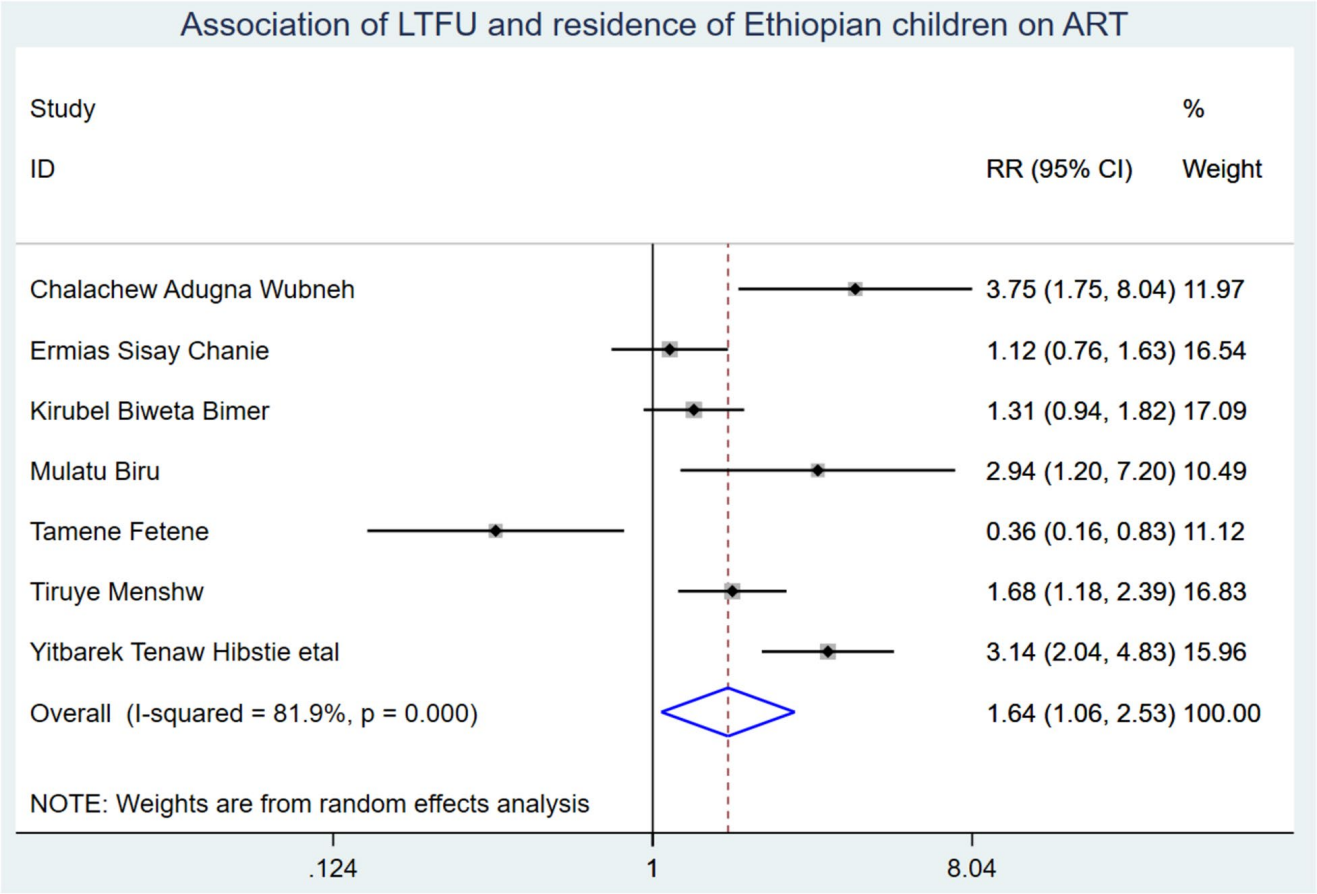
Association of LTFU and rural residency of Ethiopian children on ART

**Fig. 8:**
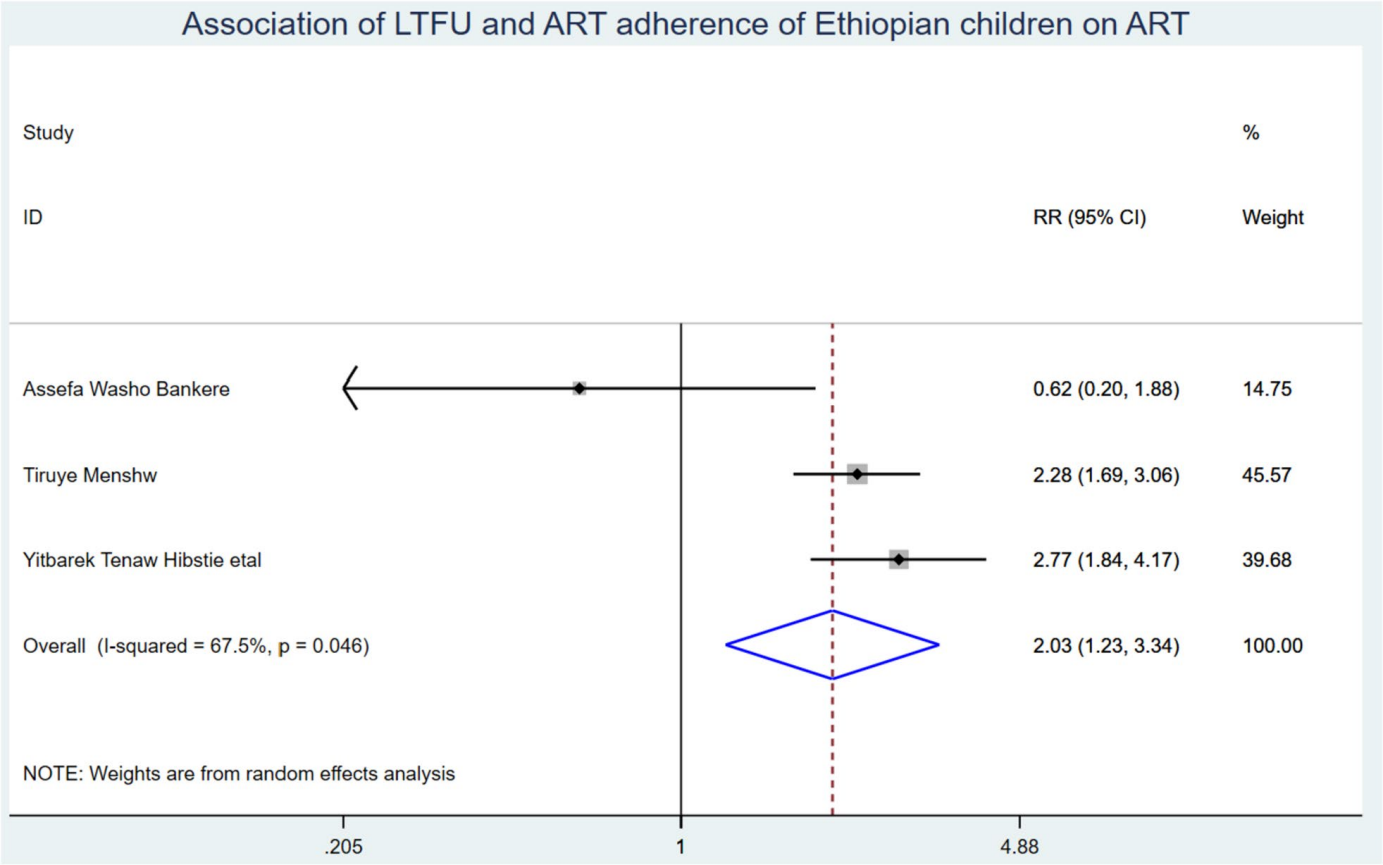
Association of LTFU and poor ART adherence among Ethiopian Children on ART

Those study participants who were from rural residences had a 1.65 (95% CI: 1.06, 2.52) times higher chance of getting LTFU when compared with those children living in urban (Fig. 7). Children who had poor ART adherence had a 2.03 (95% CI: 1.23, 3.34) times higher chance of experiencing LTFU of ART than children having good ART adherence (Fig. 8).

## Discussions

This systematic review and meta-analysis included nine articles that were published up to 2022 and had 3336 children. The sample size for each study ranged from 254 to 533 children on ART in Ethiopia. The included studies were conducted either in prospective or retrospective cohort study design and all have the same LTFU operational definition “LTFU is failing to engage in the continuum of care for 90 days (3 months) after the last scheduled appointment due to their wishes or beliefs or barriers to continued ART access for care”.

Among Ethiopian children, the pooled incidence of LTFU from ART was 5.83 (95% CI: 3.94, 7.72) per 100 children- years of observation with I^2^: 83.7% & *p*-value < 0.001. This finding is too high and requires immediate attention to accomplish the predetermined goals and targets, such as meeting the Joint United Nations Program on HIV/AIDS (UNAIDS) 95–95-95 by 2025 and ending the HIV epidemic by 2030. The high incidence of LTFU could be because most of the HIV-infected children who were experiencing LTFU were from rural and their families had low incomes, and were uneducated in their educational background. Because of the children's aforementioned circumstances, they did not receive regular ART follow-ups from the ART institution they founded. In addition to the above, due to the fear of social stigma, families may be unwilling to have regular ART follow-ups from which their children had tested and initiated ART in their locality, and those children having ART initiation and follow-up far from their locality may experience LTFU from their ART appointment schedule due to their family's economic scarcity. Further more, the children may also experience LTFU because their families had poor knowledge about the drawbacks of LTFU and regular ART follow-up practice due to their educational background plus the minister of health did not implement close follow-up and monitoring. Finally, due to the inadequate setup of ART centers for diagnosing opportunistic infections like tuberculosis, children are exposed to a double disease burden, which leads to more immunocompromization and disease progression. This ultimately results in LTFU from ART due to the development of hopelessness secondary to the disease progression. These all might be the factors associated with HIV-infected children experiencing LTFU from their ART. Here, the minister of health should be applied close follow-up like using phone message text as a reminder of children's appointment schedule, and daily pill intake for their families. Additionally, healthcare professionals should pay more attention to educating families about the importance of regular ART follow-up and the disadvantages of LTFU for children and their families.

Those children who were rural dwellers had a 1.65 (95% CI: 1.06, 2.52) times higher chance of experiencing LTFU when compared with those children who came from urban. This finding was supported by the study conducted in South Africa [38]. This could be because children from rural areas are more likely to experience loss to follow-up (LTFU) in ART centers due to several reasons. Firstly, they may be uneducated and lack economic management skills, which limits their family’s access to information and leads to low-income status. This, in turn, affects their practice on regular ART follow-up and health-seeking behavior due to the scarcity of money for transportation costs as well as the absence of nearby ART treatment centers. Secondly, the absence of integrated services for chronic diseases may also contribute to LTFU among rural dweller children. This is because it takes extra time devoted to waiting for different diagnoses and treatment services, which may lead them to have nights where money for bed and food is requested, leaving the clients unsatisfied. Therefore, it is recommended that the minister of health collaborates with governmental and non-governmental sectors to strengthen the scale-up of ART centers and initiate HIV services integrated with chronic diseases [42].

Children who have poor ART adherence had a 2.03 (95% CI: 1.23, 3.34) times higher chance of experiencing LTFU of ART than children having good ART adherence. This finding is sustained by the study conducted in Nigeria [43]. Studies conducted in Uganda have shown that rural dwelling is the primary predictor of LTFU among children on ART. This is due to several reasons. Firstly, even with regular follow-ups, children may not take their daily pills dose due to low follow-up by their families. Secondly, in rural areas, there is no matured peer support and counseling but more pronounced stigma and discrimination which leads to children developing depression and hopelessness. Thirdly, poor ART adherence leads to the progression of diseases (AIDS) called ART failure, and the development of ART-resistant viral strain narrowing the treatment option which lead to LTFU and end up with death. Therefore, it is recommended that ART centers should provide targeted adherence support for children from rural areas [44, 45]. The study’s strength lies in providing the pooled incidence and predictors of LTFU among children on ART in Ethiopia. However, it is important to note that the study only included studies that were published in English, which may be taken as the limitation of the study.

## Conclusions and recommendation

Among Ethiopian children on ART, one out of 167 children on ART had a risk of experiencing LTFU. The identified predictors of LTFU were being rural dwellers and having poor ART adherence. The authors recommend that the minister of health should strengthen the scale-up of ART centers, initiate HIV service integrated with chronic diseases, and have close follow-up and monitoring to address the high incidence of LTFU. To focus on rural dwellers, the minister of health should scale-up ART centers in collaboration with governmental and non-governmental sectors and use phone message texts as reminders of their appointment schedule. Healthcare professionals should also pay more attention to rural dwellers by providing education on the importance of having regular ART follow-ups and the drawbacks of LTFU. Children’s nearby families should control and remind the children to take their daily pill dose intake at the appropriate time and frequency. Considering poor ART adherence, healthcare professionals must ensure that children take their daily pill at the correct dose and frequency when they come for their next appointment follow-up. The minister of health should set daily reminders using phone text messages and scale-up ART centers more. Future researchers should conduct a study on the fate of children after LTFU from ART.

### Supplementary Information

**Additional file 1.**

## Data Availability

The datasets used and analyzed during the current study are available upon reasonable request from the corresponding author.

## Abbreviations

AIDS: Acquired immunodeficiency syndrome
HIV: Human Immunodeficiency Virus
UNAIDS: United Nations Program on HIV/AIDS
CLHIV: Children Living with Human Immunodeficiency Virus
LTFU: Loss to Follow-up
ART: Antiretroviral Therapy
