# Correction: Incidence and predictors of loss to follow-up among Ethiopian children on antiretroviral therapy: a systematic review and meta-analysis

**DOI:** 10.1186/s12889-024-17778-6

**Published:** 2024-01-30

**Authors:** Molla Yigzaw Birhanu, Getamesay Molla Bekele, Getasew Yirdaw, Bekele Simegn Demissie, Genanew Kassie Getahun, Selamawit Shita Jemberie

**Affiliations:** 1 Department of Public Health, College of Health Sciences, Debre Markos University, Debre Markos, P.O. Box 269, Ethiopia; 2 Department of Gynecology and Obstetric, School of Medicine, Debre Markos University, Debre Markos, Ethiopia; 3 Department of Environmental Health, College of Health Sciences, Debre Markos University, Debre Markos, Ethiopia; 4 Department of Public Health, St.Lideta College of Health Science and Business, Addis Ababa, Ethiopia; 5 Department of Public Health, Menelik II Medical and Health Science College, Addis Ababa, Ethiopia; 6 Department of Midwifery, College of Health Sciences, Debre Markos University, Debre Markos, Ethiopia

**BMC Public Health (2023) 24:169**

10.1186/s12889-023-17333-9

The original publication of this article contained an incorrect author name. The incorrect and correct information is listed in this correction article, the original article has been updated.

Incorrect:

Genanaw Kassie Getahun

Correct:

Genanew Kassie Getahun

